# miR-17-92 expression in differentiated T cells - implications for cancer immunotherapy

**DOI:** 10.1186/1479-5876-8-17

**Published:** 2010-02-18

**Authors:** Kotaro Sasaki, Gary Kohanbash, Aki Hoji, Ryo Ueda, Heather A McDonald, Todd A Reinhart, Jeremy Martinson, Michael T Lotze, Francesco M Marincola, Ena Wang, Mitsugu Fujita, Hideho Okada

**Affiliations:** 1 Department of Dermatology, University of Pittsburgh School of Medicine, 200 Lothrop Street, Pittsburgh, PA, 15213, USA; 2 Department of Immunology, University of Pittsburgh School of Medicine, 200 Lothrop Street, Pittsburgh, PA, 15213, USA; 3 Department of Neurological Surgery, University of Pittsburgh School of Medicine, 200 Lothrop Street, Pittsburgh, PA, 15213, USA; 4 Department of Surgery, University of Pittsburgh School of Medicine, 200 Lothrop Street, Pittsburgh, PA, 15213, USA; 5 Brain Tumor Program, University of Pittsburgh Cancer Institute, G12a Hillman Cancer Center, 5117 Centre Ave, Pittsburgh, PA, 15213, USA; 6 Department of Infectious Diseases and Microbiology, University of Pittsburgh Graduate School of Public Health, A419 Crabtree Hall, Pittsburgh, PA 15261, USA; 7 National Institutes of Health, Department of Transfusion Medicine, Building 10, Room 1C711, Clinical Center, Bethesda, MD 20892, USA

## Abstract

**Background:**

Type-1 T cells are critical for effective anti-tumor immune responses. The recently discovered microRNAs (miRs) are a large family of small regulatory RNAs that control diverse aspects of cell function, including immune regulation. We identified miRs differentially regulated between type-1 and type-2 T cells, and determined how the expression of such miRs is regulated.

**Methods:**

We performed miR microarray analyses on *in vitro *differentiated murine T helper type-1 (Th1) and T helper type-2 (Th2) cells to identify differentially expressed miRs. We used quantitative RT-PCR to confirm the differential expression levels. We also used WST-1, ELISA, and flow cytometry to evaluate the survival, function and phenotype of cells, respectively. We employed mice transgenic for the identified miRs to determine the biological impact of miR-17-92 expression in T cells.

**Results:**

Our initial miR microarray analyses revealed that the miR-17-92 cluster is one of the most significantly over-expressed miR in murine Th1 cells when compared with Th2 cells. RT-PCR confirmed that the miR-17-92 cluster expression was consistently higher in Th1 cells than Th2 cells. Disruption of the IL-4 signaling through either IL-4 neutralizing antibody or knockout of signal transducer and activator of transcription (STAT)6 reversed the miR-17-92 cluster suppression in Th2 cells. Furthermore, T cells from tumor bearing mice and glioma patients had decreased levels of miR-17-92 when compared with cells from non-tumor bearing counterparts. CD4^+ ^T cells derived from miR-17-92 transgenic mice demonstrated superior type-1 phenotype with increased IFN-γ production and very late antigen (VLA)-4 expression when compared with counterparts derived from wild type mice. Human Jurkat T cells ectopically expressing increased levels of miR-17-92 cluster members demonstrated increased IL-2 production and resistance to activation-induced cell death (AICD).

**Conclusion:**

The type-2-skewing tumor microenvironment induces the down-regulation of miR-17-92 expression in T cells, thereby diminishing the persistence of tumor-specific T cells and tumor control. Genetic engineering of T cells to express miR-17-92 may represent a promising approach for cancer immunotherapy.

## Background

We have focused on the development of effective immunotherapeutic strategies for central nervous system (CNS) tumors, such as glioblastoma multiforme (GBM). Preclinical studies have demonstrated that tumor-specific T helper type-1 (Th1) and T cytotoxic type-1 (Tc1) cells, but not type-2 counterparts, can efficiently traffic into CNS tumor sites and mediate effective therapeutic efficacy, recruited via the type-1 chemokine CXCL10 [1-3] and the integrin receptor, Very Late Antigen (VLA)-4 [4-7]. Despite the importance of the type-1 T cell response, cancers, including GBMs, secrete numerous type-2 cytokines [8-10] that promote tumor proliferation [11,12] and immune escape [13]. Hence, the strategic skewing of existing type-2 to type-1 immunity in glioma patients may be critical for the development of more effective immunotherapy.

MicroRNAs (miRs) are a novel class of endogenous small single-stranded RNA molecules which are 18-24 nucleotides in length [14]. Mature miRs repress mRNA encoded protein translation and are highly conserved between species, including viruses, plants and animals [15]. There are over 700 miRs identified in the human genome that collectively are predicted to regulate two-thirds of all mRNA transcripts [14]. Findings over the past several years strongly support a role for miRs in the regulation of crucial biological processes, such as: cellular proliferation [16], apoptosis [17], development [18], differentiation [19], metabolism [20], and immune regulation [21,22]. We recently reported that miR-222 and -339 in cancer cells down-regulate the expression of an intercellular cell adhesion molecule (ICAM)-1, thereby regulating the susceptibility of cancer cells to cytotoxic T lymphocytes (CTLs) [23]. This is among the first reports to demonstrate the role of miR in cancer immunosurveillance.

In the current study, in an effort to understand the potential roles of miRs in anti-tumor immunity, we examined miRs differentially expressed in Th1 and Th2 cells. Our miR microarray and RT-PCR analyses revealed that of all analyzed miRs, members of the miR-17-92 cluster (miR-17-92) are of the most significantly over-expressed miRs in murine Th1 cells when compared with Th2 cells. The miR-17-92 transcript encoded by mouse chromosome14 (and human chromosome 13) is the precursor for 7 mature miRs (miR-17-5p, miR-17-3p, miR-18a, miR-19a, miR-20a, miR-19b and miR-92) [24,25]. This cluster is also homologous to the miR-106a-363 cluster on the X chromosome and the miR-106b-25 cluster on chromosome 5. Together, these three clusters contain 15 miR stem-loops, giving rise to 14 distinct mature miRs that fall into 5 miR families. The members in each family have identical seed regions. This genomic organization is highly conserved in all vertebrates for which complete genome sequences are available [26].

miRs in the miR-17-92 cluster are amplified in various tumor types, including B cell lymphoma and lung cancer, and promote proliferation and confer anti-apoptotic function in tumors, thereby promoting tumor-progression [27-31]. Knockout and transgenic studies of the miR-17-92 cluster in mice have demonstrated the importance of this cluster in mammalian biology [25]. Transgenic mice with miR-17-92 overexpressed in lymphocytes develop lymphoproliferative disorder and autoimmunity but not cancer [24]. These findings demonstrate a critical role for miR-17-92 cluster in T cell biology.

We show here that miR-17-92 is up-regulated in Th1 cells when compared with Th2 cells. IL-4 and STAT6 signaling mediate the down-regulation of miR-17-92. Tumor-bearing host conditions also suppress the miR-17-92 cluster expression in T cells, which is associated with a loss in ability to produce IFN-γ. This led us to hypothesize that miR-17-92 cluster overexpression might enhance type-1 responses. Indeed, type-1 T cells derived from miR-17-92 transgenic mice demonstrated a more pronounced type 1 phenotype including enhanced IFN-γ production and increased VLA-4 expression when compared with control type-1 T cells. These findings suggest that miR-17-92 plays a critical role in type-1 adaptive immunity.

## Materials and methods

### Reagents

RPMI 1640, FBS, L-glutamine, sodium pyruvate, 2-mercaptoethanol, nonessential amino acids, and penicillin/streptomycin were obtained from Invitrogen Life Technologies. Recombinant murine (rm) IL-12 was purchased from Cell Sciences Technologies. RmIL-4, recombinant human (rh) IL-4 and rhIL-2 were purchased from PeproTech. Purified monoclonal antibodies (mAbs) against IL-12 (C15.6), IFN-*γ *(R4-6A2), IL-4 (11B11), CD3 (145-2C11), CD4 (RM4-5), CD8 (53-6.7) and CD49d (R1-2) were all purchased from BD Pharmingen. Purified mAbs against CD3 (UCHT1) and CD28 (CD28.2) and IL-4 (MP4-25D2) were purchased from Biolegend. RT-PCR reagents and primers were purchased from Applied Biosystems and analyzed on a BioRad IQ5. WST-1 reagent was purchased from Roche. For isolation of T cells, immunomagenic isolation kits from Miltenyi Biotec were used. All reagents and vectors for lentiviral production were purchased from System Biosciences with the exception of Lipofectamine 2000, which was from Invitrogen.

### Mice

C57BL/6 mice and C57BL/6 background STAT6 deficient mice (B6.129S2 [C]-*Stat6*^*tm*1*Gru*^/J; The Jackson lab) (both 5-9 wk of age) were purchased from The Jackson Laboratory. C57BL/6-background miR-17-92 transgenic (TG) mice (C57BL/6-*Gt [ROSA]26Sor*^*tm*3(*CAG*-*MIRN*17-92,-*EGFP*)*Rsky*^/J; The Jackson Lab) were maintained in the Hillman Cancer Center Animal Facility at University of Pittsburgh as breeding colonies and bred to C57BL/6-background mice transgenic for *Cre *recombinase gene under the control of the *Lck *promoter (B6.Cg-Tg [Lck-cre]548Jxm/J, the Jackson Lab) to obtain mice, in which T cells expressed miR-17-92 at high levels (miR-17-92 TG/TG). For mouse tumor experiments, C57BL/6 mice and C57BL/6 background *STAT6*^-/- ^mice received subcutaneous injection of 1 × 10^6 ^B16 tumor cells resuspended in PBS into the right flank. On day 15 following tumor inoculation, mice were sacrificed and splenic T cells were isolated. Animals were handled in the Hillman Cancer Center Animal Facility at University of Pittsburgh per an Institutional Animal Care and Use Committee-approved protocol.

### T cells from Healthy Donors and Patients with GBM

This study was approved by the local ethical review board of University of Pittsburgh. All healthy donors and patients with GBM signed informed consent before blood samples were obtained. To determine the impact of IL-4, healthy donor-derived CD4^+ ^T cells were isolated with immunomagentic-seperation and stimulated with 100 IU/ml rhIL-2, anti-CD3 and anti-CD28 mAbs (1 μg/ml for each) in the presence or absence of rhIL-4(10 ng/ml). RT-PCR analyses were performed with both healthy donor- and patient-derived T cells to determine the expression of miR-17-92 as described in the relevant section.

### Th1 and Th2 Cell Culture

Th1 and Th2 cells were differentiated from immunomagnetically-separated CD4^+ ^splenic T cells. Magnetic activated cell separation (MACS) was carried out using positive selection. Briefly, spleens were minced in complete media, resuspended in red blood cell lysis buffer and stained with immunomagnetically labeled anti-CD4 antibody. Cells were then washed and placed through the magnetic column in 500 μl of MACS buffer. The column was then washed 3 times with buffer and then removed from the magnet and labeled cells were extracted in 3 ml of MACS buffer.

For differentiation of T cells, purified CD4^+ ^cells were stimulated in 48 well plates with anti-CD3 mAb (5 μg/ml) in the presence of irradiated C57BL/6 spleen cells (3000 Rad) as feeder cells. RmIL-12 (4 ng/ml), rmIFN-γ (4 ng/ml), anti-IL-4 (10 μg/ml) mAb and rhIL-2 (100 IU/ml) were added for Th1 development. Th2 cells were generated from the same CD4^+ ^cell precursors stimulated with anti-CD3 mAb and feeder cells in the presence of rmIL-4 (50 ng/ml), two anti-IFN-γ mAbs (10 μg/ml), anti-IL-12 mAb (10 μg/ml) and rhIL-2 (100 IU/mL). After 10 days cells were stained for IL-4 and IFN-γ to confirm differentiation. Neutral cell culture included anti-CD3, feeder cells and rhIL-2. For studies involving IL-4 blockade, 12.5 ng/ml anti-human IL-4 mAb (Biolegend) was used in human experiments and 2.5 μg/ml anti-mouse IL-4 mAb (11B11) in murine studies. IFN-γ and IL-4 in the culture supernatants were measured using specific ELISA kits (R&D Systems). For FACs analysis, cells were incubated with mAb at 4°C for 30 min, washed twice in staining buffer, and fixed in 500 μl of 2% paraformaldehyde in PBS. Cells were stored in the dark at 4°C until analysis. Flow cytometry was carried out on the Coulter XL four-color flow cytometer at the flow cytometry core facility of the University of Pittsburgh Cancer Institute.

### miR Microarray

Total RNA was isolated from Th1 and Th2 cells using the Trizol reagent and quality was confirmed with an A260/A280 ratio greater than 1.85. Two μg of total RNA was labeled with either Hy5 (red; Th1) or Hy3 (green; Th2) fluorescent dyes using miRCURY LNA microRNA labeling kit (Exiqon, Woburn, MA) according to manufacturer's protocol. Labeled miR samples in duplicate were cohybridized on to miR array slides, a custom spotted miR array V4P4 containing duplicated 713 human, mammalian and viral mature antisense microRNA species (miRBase: http://microrna.sanger.ac.uk/, version 9.1) plus 2 internal controls with 7 serial dilutions printed in house (Immunogenetics Laboratory, Department of Transfusion Medicine, Clinical Center, National Institutes of Health) [32]. After washing, raw intensity data were obtained by scanning the chips with GenePix scanner Pro 4.0 and were normalized by median over entire array. Differentially expressed miRs were defined by mean (n = 2) fold change (Th1/Th2 signal intensity) >2.

### Quantitative RT-PCR

Total RNA was extracted using the Qiagen RNeasy kit and quality was confirmed with a A260/A280 ration greater than 1.85. RNA was subjected to RT-PCR analysis using the TaqMan microRNA Reverse Transcription Kit, microRNA Assays (Applied Biosystems), and the Real-Time thermocycler iQ5 (Bio-Rad). The small nucleolar SNO202 was used as the housekeeping small RNA reference gene for all murine samples and RNU43 for human samples. All reactions were done in triplicate and relative expression of RNAs was calculated using the ΔΔ*C*_T _method [33].

### WST-1 Proliferation Assay

For WST-1 proliferation assays, 1 × 10^4 ^cells were cultured in a 96 well plate for 24-48 hours in 100 μl of complete media. Then, 10 μl of WST-1 reagent was added to each well. Cells were incubated at 37°C, 5% CO_2 _for 4 hours, and placed on a shaker for 1 min. The plates were then read on a micro plate reader with a wavelength of 420 nm and a reference at 620 nm.

### Assays using Jurkat lymphoma cells transduced with miR-17-92

Jurkat human T cell leukemia cells (American Type Culture Collection) were transduced by either one of the following pseudotype lentiviral vectors: 1) control vector encoding GFP; 2) the 17-92-1 expression vector encoding miR-17 18 and 19a, or 3) the 17-92-2 expression vector encoding miR 20, 19b-1, and 92a-1. All vectors were purchased from SBI. Lentiviral particles were produced by co-transfecting confluent 293TN cells (SBI) with pPACK-H1 Lentivirus Packaging Kit (SBI) and the miR containing expression vectors (SBI) noted above using Lipofectamine 2000 reagent (Invitrogen). Supernatant was collected after 48 hour incubation at 37°C with 5% CO_2 _and placed at 4°C with PEG-it Virus Concentration Solution (SBI) for 24 hrs. Supernatants/PEG solutions were then centrifuged and the pellet was resuspended in a reduced volume of media as viral stock. Jurkat cells were further resuspended in the viral stock together with polybrene (8 μg/ml) for 24 hrs. Fresh media was then added to the cells and transduction efficiency was evaluated by GFP expressing cells. For IL-2 production, transduced Jurkat cells were stimulated with Phorbol 12-myristate 13-acetate (PMA) (10 ng/ml) and ionomycin (500 nM) for overnight and supernatant was assayed for IL-2 by a human IL-2 ELIZA kit. For activation induced cell death (AICD), cells were treated with 10 μg/ml purified anti-CD3 mAb (UCHT1) from Biolegend for 24 hours and then cell viability was measured using WST-1 reagent.

### Statistical Methods

All statistical analyses were carried out on Graphpad Prism software. The statistical significance of differences between groups was determined using student t- test. We considered differences significant when *p *< 0.05. A post test for linear trend test was used to determine linear trend and we considered *p *< 0.05 to be significant.

## Results

### miR-17-92 and its paralogs are overexpressed in Th1 cells compared with Th2 cells

**Figure 1:**
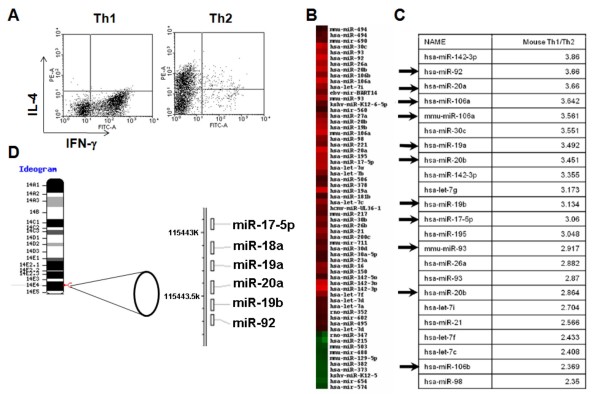
**Microarray analysis demonstrates up-regulation of miR-17-92 in Th1 cells**. **(A)**, Intracellular IFN-γ vs. IL-4 expression of Th1 and Th2 cells induced from mouse CD4^+ ^splenic T cells *in vitro*. **(B)**, Differentially expressed miRs were analyzed by hierarchical clustering of the log2 value of Th1/Th2 pair of miR microarray signal. Red indicates up-regulation in Th1; green, up-regulation in Th2. **(C)**, miRs were ranked by relative fold expression in Th1/Th2 cells. Arrows indicate members of the miR-17-92 cluster or paralog clusters. miRs with a relative expression of >2.35 fold in Th1 are shown. **(B and C)**, hsa- and mmu- indicate human and mouse miR probes, respectively. Hsa-probes can hybridize with most mouse miR due to the high homology and mmu-signals are shown only when murine miR has unique sequence compared to its human counterpart. **(D)**, Ideogram of mouse chromosome 14 showing the location and order of the miR-17-92 cluster (adapted from NCBI Blast).

**Figure 2:**
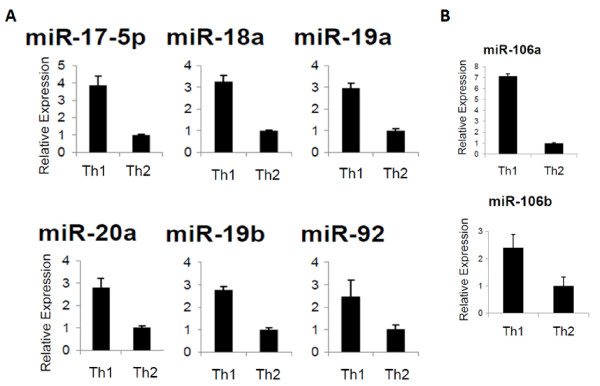
**Enhanced expression of miRs from the miR-17-92 cluster in Th1 cells**. Data represent relative expression of mature miRs in Th1 compared with Th2 cells. *SNO202 *was used as the internal control and ΔΔ*C*_T _method was used to examine expression relative to the Th2 cell value. Relative expression is shown for **(A)**, miR-17-92 cluster members or **(B)**, representative paralog cluster members, miR-106a and -106b. Error Bars indicate standard deviation of the triplicate samples. Each experiment was repeated at least 3 times. Up-regulation in Th1 vs. Th2 is significant in **(A) **with p < .01 for miR-92 and p < .0001 for all other miRs and in **(B)**, with p < .001 for miR-106a and p < .05 for miR106b using the student t test.

To identify differentially expressed miRs between Th1 and Th2 cells, we performed a miR microarray analysis. From mouse splenic CD4^+ ^T cells, Th1 and Th2 cells were generated as described in Materials and Methods. These T cells exhibited expected cytokine profiles with Th1 cells dominantly producing IFN-γ but not IL-4, while Th2 cells produce mostly IL-4 (Fig. 1A). Total RNA was extracted from these T cells, and analyzed for differential miR expression by miR microarray for 714 miRs (Fig. 1B). Hierarchical clustering of differentially expressed miRs revealed distinct miR expression profiles between the Th1 and Th2 cells. Eleven of the miRs from the miR-17-92 cluster and its paralogs were expressed at higher levels in Th1 cells than in Th2 cells. Next, we ranked the miRs preferentially expressed in Th1 cells according to the fold difference of expression when compared with Th2 cells (Fig. 1C). Interestingly, members of miRs in the miR-17-92 clusters were identified as the most differentially expressed of all miRs in Th1 cells compared to Th2 cells. Since miR-17-92 clusters appear to be transcribed as single polycistronic transcripts (Fig. 1D), we expected that all the miRs from the miR-17-92 cluster would be consistently expressed at higher levels in Th1 cells than in Th2 cells, which was confirmed by RT-PCR analysis (Fig. 2A).

The miR-17-92 cluster has 2 paralog clusters: miR-106a-363 and miR-106b-25. These paralog clusters target similar mRNAs as the miR-17-92 cluster due to high sequence homology [34]. To establish if these paralog miR clusters are also overexpressed in our Th1 vs. Th2 cells, we next performed RT-PCR for miRs in each of these clusters. Representative for these paralog clusters are miR-106a and miR106b (Fig. 2B). These data demonstrate that the paralog clusters of miRs were also up-regulated in Th1 cells over Th2.

### Neutralization of endogenous IL-4 up-regulates miR-17-92 cluster miRs in T-cells

**Figure 3:**
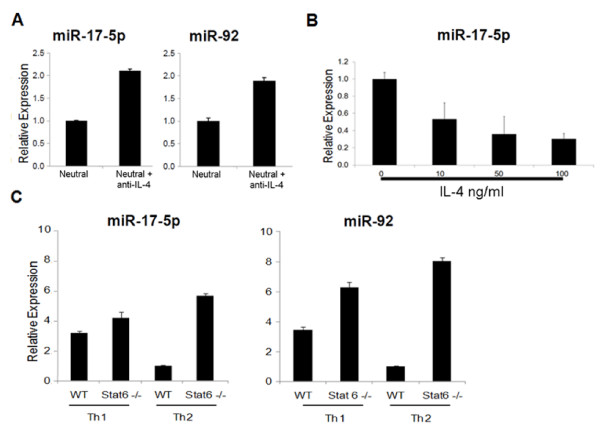
**Modulation of miR-17-92 expression by IL-4 signaling**. **(A) **Immuno-magnetically isolated mouse splenic CD4^+ ^T cells were cultured with 5 μg/ml plated anti-CD3, feeder cells and 100 U/ml hIL-2 ("Neutral" condition). Anti-IL-4 (2.5 μg/ml) or isotype control mAb was added to the appropriate wells and cultured for 5 days prior to extraction of total RNA. Statistical analysis was carried out using the student t test. The blockade of IL-4 up-regulated miR-17-5p and miR-92 significantly with p < .001 and p < .005, respectively. **(B)**, CD4^+ ^T cells were cultured with anti-CD3, feeder cells, and hIL-2 and varying amounts of IL-4 for 5 days. Total RNA was extracted and analyzed by RT-PCR for miR-17-5p expression. The dose dependent decrease of miR-17-92 expression was analyzed using post test for linear trend and was significant (p < .001). **(C)**, Th1 and Th2 cells were induced from splenic CD4^+ ^T cells isolated from either wild-type or *STAT6*^-/- ^mice. Total RNA was extracted and RT-PCR was performed using specific primers against miR-17-5p and miR-92. Columns represent the mean of triplicates from one of 2 two experiments with similar results, and error bars represent standard deviations. *STAT6*^-/- ^cells demonstrated significantly higher levels of miR-17-5p and miR-92 compared with wild type (WT) cells in both Th1 and Th2 conditions (*p *< .001) using the student t test.

In order to identify factors that contribute to the differential expression of miR-17-92 cluster miRs between Th1 and Th2 cells, we next sought to determine whether a prototypical type-2 inducing cytokine, IL-4, would affect miR-17-92 expression in CD4^+ ^T cells. Neutralization of endogenous IL-4 by specific mAb against IL-4 up-regulated miR-17-92 cluster miRs in CD4^+ ^T cells stimulated with IL-2 without addition of Th1-inducing factors IL-12 or IFN-γ, by approximately 50% (Fig. 3A). The anti-IL-4 mAb also up-regulated miR-17-92 in Th2 culture conditions as well (data not shown). To determine whether there is an IL-4 dose-dependent suppression of miR-17-92 cluster, we next treated CD4^+ ^T cells with increasing doses of IL-4 at 0, 10, 50 or 100 ng/ml and measured miR-17-5p expression by RT-PCR (Fig. 3B). miR-17-92 suppression was a dose-dependent phenomenon.

### Up-regulated miR-17-92 expression in STAT6 deficient T cells

To further elucidate the effect of IL-4 signaling on miR-17-92 cluster expression, we next cultured CD4^+ ^T cells under Th1 or Th2 skewing conditions from mice deficient of the critical IL-4 signaling molecule, STAT6 [4,35]. Both Th1 and Th2 cultured cells induced from STAT6-deficient mice showed higher levels of miR-17-5p expression compared with corresponding WT Th cells, suggesting a novel critical role of IL-4R/STAT6-signaling in the down-regulation of miR-17 expression (Fig. 3C).

### Suppression of miR-17-92 may occur in cancer-bearing hosts

**Figure 4:**
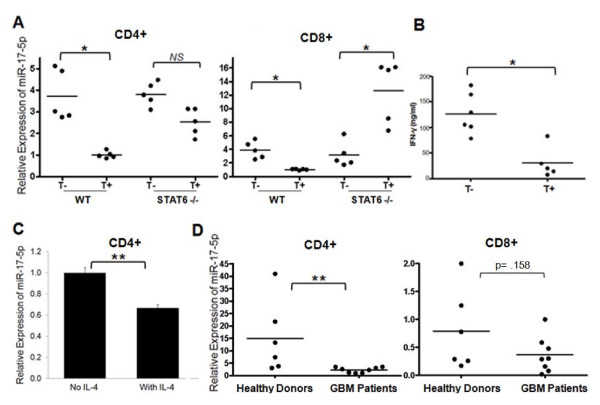
**Tumor bearing conditions down-regulate miR-17-5p expression in T cells**. SPCs were harvested from C57BL/6 or *STAT6*^-/- ^mice bearing day 15 subcutaneous B16 melanoma (T+) or control non-tumor bearing mice (T-). **(A)**, CD4^+ ^and CD8^+ ^T cells were isolated by immuno-magnetic bead separation, and evaluated for miR17-5p expression. **(B)**, 1 × 10^6 ^CD4^+ ^cells from WT mice were briefly stimulated with anti-CD3 mAb for 6 hours. Concentration of IFN-γ secreted in culture media was evaluated by specific ELISA. **(C)**, CD4^+ ^T cells were isolated from healthy donor-derived peripheral blood mononuclear cells (PBMC) and stimulated with 5 μg/ml plated anti-CD3, feeder cells (irradiated PBMC) and 100 IU/ml hIL2 in the presence or absence of hIL-4 (10 ng/ml) for 5 days prior to extraction of total RNA. **(D)**, Non-stimulated CD4^+ ^and CD8^+ ^T cells were isolated by immuno-magnetic beads from PBMC derived from healthy donors (n = 6) or patients with GBM (n = 8) and miR-17-5p expression was analyzed by RT-PCR. Data in **(A)**, **(B) **and **(C)**, are representative of 2 identical experiments with similar results. Columns represent the mean of triplicates from a single experiment and error bars represent standard deviation. * indicates *p *< 0.01 and ** indicates p < 0.05 between the two groups using the student t test.

These data led us to hypothesize that suppression of miR-17-92 would occur in cancer-bearing hosts where tumor-derived factors likely promote Th2-skewed immune responses and secretion of IL-4 [8]. Indeed, CD4^+ ^and CD8^+ ^splenocytes (SPCs) derived from wild type C57BL/6 mice bearing B16 subcutaneous tumors expressed lower levels of miR-17-5p when compared with those derived from non-tumor bearing mice (Fig. 4A). Interestingly, the tumor bearing condition did not suppress miR-17-5p expression by CD4^+ ^T cells in *STAT6*^-/- ^mice. Furthermore, CD8^+ ^T cells in *STAT6*^-/- ^mice demonstrated enhanced levels of miR-17-5p expression when these mice bore B16 tumors compared with non-tumor bearing mice. When wild type CD4^+ ^T cells were stimulated with anti-CD3 mAb *in vitro *for 24 hours, the CD4^+ ^T cells from tumor-bearing mice produced lower levels of IFN-γ when compared with ones from non-tumor bearing wild type mice (Fig. 4B). These data suggest that tumor-associated immunosuppression may involve the down-regulation of miR-17-92 through a STAT6 dependant pathway.

We next evaluated whether the observed IL-4-mediated and tumor-induced suppression of miR-17-92 are relevant in human T cells. When healthy donor-derived CD4^+ ^T cells were stimulated with rhIL-2, anti-CD3 and anti-CD28 mAbs, consistent with the mouse data, addition of rhIL-4 in the cultures suppressed expression of miR-17-5p (Fig. 4C). Moreover, CD4^+ ^T cells obtained from patients with GBM exhibited significantly decreased levels of miR-17-5p when compared with ones from healthy donors (Fig. 4D). Thus both IL-4 and GBM-bearing conditions suppress miR-17-5p expression in CD4^+ ^T cells. Although not statistically significant, CD8^+ ^T cells demonstrated a trend towards decreased levels of miR-17-5p expression in GBM patients when compared with healthy donors (Fig. 4D).

### T cells derived from miR-17-92 transgenic mice display enhanced type-1 phenotype

**Figure 5:**
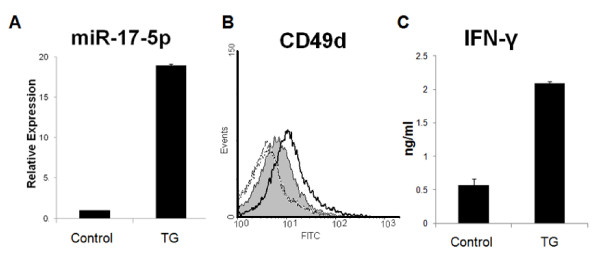
**T cells from miR-17-92 transgenic mice demonstrate enhanced Th1 phenotype**. Splenic CD4^+ ^T cells were immuno-magnetically isolated from miR-17-92 TG/TG or control animals. **(A)**, miR-17-5p expression was analyzed in total RNA extracted from these freshly isolated cells. **(B)**, Flow analysis was carried out on these freshly isolated cells for surface expression of CD49d, a subunit composing VLA-4. The grey-shaded region represents CD4^+ ^T cells isolated from control wild type animals and the unshaded region with the solid line represents CD4^+ ^T cells from miR-17-92 TG/TG mice. Dotted lines represent samples stained with isotype control Rat IgG2b. As the background staining with the isotype IgG2b was equally very low in the two cell types, the corresponding histograms are barely distinguishable each other. **(C)**, Isolated cells were stimulated in Th1 skewing condition for 9 days and 5 × 10^6^cells were then plated in fresh media for 24 hours, at which point supernatant was collected and analyzed for IFN-γ by ELISA. Both in **(A)**, and **(C)**, values in the two groups were statistically different with p < .01 using the student t test.

The data discussed above strongly suggest GBM-associated factors and a type-2 promoting cytokine (IL-4) down-regulate miR-17-92 in T cells. miR-17-92 is expected to play pivotal roles in T cell functions. We therefore sought to determine whether ectopic expression of miR-17-92 would promote the type-1 phenotype of T cells. As detailed in Materials and Methods, we produced mice that overexpress miR-17-92 specifically in T cells (miR-17-92 TG/TG). We isolated CD4^+ ^splenocytes from these mice and evaluated the expression of miR-17-5p (Fig. 5A). CD4^+ ^cells from TG/TG mice displayed a greater than 15 fold increase in miR-17-p5 expression as compared with controls. These cells also expressed elevated levels of CD49d, which is a subunit composing a type-1 T cell marker VLA-4 (Fig. 5B). Although CD49d (also known as α4-integrin) can form heterodimers with both β1 (CD29) and β7 integrins, α4β7 complexes were not expressed by either Th1 cells or Th2 cells, suggesting that CD49d is a suitable surrogate for VLA-4 expression levels [4-7]. miR-17-92-TG/TG CD4^+ ^cells also demonstrated enhanced ability to produce IFN-γ upon stimulation (Fig. 5C). Similar data were obtained with CD8^+ ^T cells isolated from these TG/TG mice (data not shown). These findings suggest that miR-17-92 promotes the type-1 phenotype in differentiating T cells.

### Ectopic expression of miR-17-92 promotes IL-2 production and resistance against activation-induced cell death (AICD) in Jurkat cells

**Figure 6:**
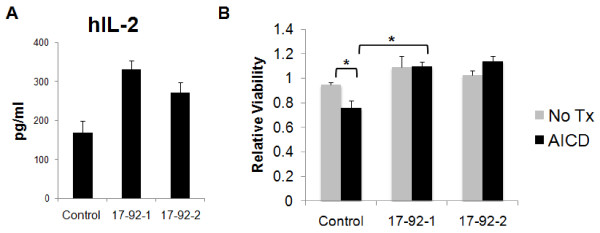
**Ectopic expression of miR-17-92 cluster members in the human Jurkat T cell line confers increased IL-2 production and resistance to AICD**. Jurkat cells were transduced by either one of the following pseudo typed lentivirus vectors: 1) control vector encoding GFP; 2) the 17-92-1 expression vector encoding miR-17 18 and 19a, or 3) the 17-92-2 expression vector encoding miR 20, 19b-1, and 92a-1. **(A)**, Transduced Jurkat cells (5 × 10^4^) in the triplicate wells were stimulated with PMA (10 ng/ml) and ionomycin (500 nM) for overnight and supernatant was harvested and tested for the presence of IL-2 by specific ELISA. The figure shows mean values and standard deviations of the amount of IL-2 released from each group. Statistical analysis was carried out using the student t test, and significant (p < .005) increase of IL-2 production was confirmed in both 17-92-1 and the 17-92-2 transduced groups compared with the control group. **(B)**, Transduced Jurkat cells were treated with the AICD inducing condition (10 μg/ml anti-CD3 mAb) or in complete media (No Tx) for 24 hrs. Then, the relative numbers of viable cells were evaluated by 4 hour WST-1 assays. The figure shows mean values and standard deviations of 8 wells/group each containing 5 × 10^5 ^cells. For each group, the relative OD readings at 450 nm of AICD-treated cells compared with control Jurkat cells without AICD-treatment is indicated. * indicates p < 0.05 between the two groups using student t test.

miR-17-92 is expected to play pivotal roles in T cell survival as well as functions. To evaluate these aspects, we transduced Jurkat cells with lentiviral vectors encoding green fluorescence protein (GFP) and either the miR-17-92-1 expression vector encoding miR-17 18 and 19a, or the 17-92-2 expression vector encoding miR 20, 19b, and 92. The control vector encodes GFP, but not miRs. Transduced Jurkat cells were stimulated with PMA and ionomycin for overnight before the supernatants were assayed for IL-2 production by ELISA (Fig. 6A). Transduction of either miR-vector promoted IL-2 production in Jurkat cells.

AICD and chemotherapy-induced suppression of T cells represent major obstacles for efficient T cell-based cancer immunotherapy [36,37]. We next examined whether transfection of Jurkat cells with miR-17-92 confers T cells resistant to AICD. AICD was induced by cultivation of Jurkat cells in the presence of 10 μg/ml anti-CD3 mAb, which is hyper-stimulatory and used as a standard method to induce AICD [38]. As demonstrated in (Fig. 6B), the growth of control Jurkat cells was significantly suppressed by nearly 25% in the AICD inducing condition compared with the same cells with the regular (growth-promoting) dose of anti-CD3 mAb (1 μg/ml). In contrast, the growth of Jurkat cells transduced with either miR-17-92-1 or miR-17-92-2 was not significantly altered by the high dose (10 μg/ml) of anti-CD3 mAb, suggesting that the miR-17-92 transfection confers T cells with substantial resistance against AICD. These findings point to a potential utility for miR-17-92 transfected T cells in cancer immunotherapy.

## Discussion

Attaining effective tumor immunity is a major goal of modern biologic therapy, limited by the tumor microenvironment and profound regulatory mechanisms limiting T cell and NK cell effectors. Here we show that the type-2-skewing tumor microenvironment induces down-regulation of miR-17-92 expression in T cells, thereby hampering anti-tumor T cell responses. It also suggests that development of immunotherapy using miR-17-92-transduced T cells is warranted based on our findings demonstrating that ectopic expression of miR-17-92 in T cells leads to improved type-1 functions, including increased VLA-4 expression and IFN-γ production.

Blockade of endogenous IL-4 by inhibitory mAb or disruption of STAT6 signaling was sufficient to up-regulate miR-17-92 in T cells (Fig 3). These findings suggest that STAT6 may negatively regulate miR-17-92 expression in T cells. Several transcription factors have been identified that regulate expression of this miR cluster, including the E2 transcription factor (E2F) family members [39,40], c-Myc [41], STAT3 [42], as well as the sonic hedgehog pathway [43,44]. How IL-4 and the STAT6 signaling pathway negatively influence miR-17-92 expression at a molecular level remains to be elucidated. With regard to the effects of IL-4/STAT6 signaling on Th1 vs. Th2 functions, we have recently demonstrated that STAT6-/- Th2 cells exhibit Th1 phenotype with increased surface expression of VLA-4 [45]. These observations have led us to hypothesize that STAT6-regulated miR-17-92 may contribute to the promotion of type-1 T cell functions.

Our findings indicate that the tumor-bearing host down-regulates miR-17-92 in T cells (Fig. 3 and 4). Interestingly, not only are *STAT6*^-/- ^T cells resistant to tumor-induced inhibition of miR-17-5p, but CD8^+ ^T cells in tumor bearing *STAT6*^-/- ^mice exhibited higher levels of miR-17-5p when compared with CD8^+ ^T cells obtained from non-tumor bearing *STAT6*^-/- ^mice. In addition to IL-4, other tumor-derived factors are likely to be involved in these events. Further studies are warranted to elucidate the molecular mechanisms underlying the regulation of miR-17-92 in T cells, especially in the tumor microenvironment.

While tumor bearing mice demonstrated decreased levels of miR-17-92 in both CD4^+ ^and CD8^+ ^cells, human GBM patients exhibited a statistically significant decrease of miR-17-92 in CD4^+ ^cells but not in CD8^+ ^cells (Fig. 4). However, there still appears to be a trend towards lower miR-17-92 expression in GBM patient-derived CD8^+ ^cells compared with those obtained from healthy donors. The lesser degree of miR-17-92 suppression in CD8^+ ^cells compared with CD4^+ ^cells in GBM patients is plausible based on our current understanding of CD4^+ ^and CD8^+ ^T cell biology. The type-1 vs. type-2 differentiation appears to be more distinct for CD4^+ ^T cells than for CD8^+ ^cells [46,47], and this may also be the case for miR-17-92. Another speculation is that CD8^+ ^T cells may be less sensitive to IL-4 than CD4^+ ^T cells thereby exhibiting less repression of miR-17-92. Further studies with larger sample size are warranted.

Messages encoding proteins that are targeted by miR-17-92 cluster miRs include: E2F1, E2F2, E2F3 [40,41], P21 [48], anti-angiogenic thrombospondin-1 and connective tissue growth factor [49], proapoptotic Bim, and phosphatase and tensin homolog (PTEN) [24]. These proteins are all involved in cell cycle regulation or apoptotic cell death, further supporting the importance of miR-17-92 cluster in T cell biology. In fact, Bim and PTEN are down-regulated in T cells overexpressing miR-17-92 [24]. Furthermore, TGF-β receptor II (TGFBRII) is one of the established targets of miR-17-92 [50]. We are currently evaluating whether miR-17-92 transgenic T cells show down-regulated TGFBRII and decreased sensitivity to TGF-β.

In agreement with others [24], our findings demonstrating increased IFN-γ production from miR-17-92 TG/TG T cells compared with control cells suggest that miR-17-92 may actually promote the type-1 skewing of T cells (Fig. 5 and 6C). As miR-17-92 targets hypoxia-inducible factor (HIF)-1α in lung cancer cells [51], enhanced miR-17-92 expression in activated T cells may promote the type-1 function of T cells at least partially through down-regulation of HIF-1α. Although HIF-1 expression provides an important adaptation mechanism of cells to low oxygen tension [52,53], it does not appear to be critical for survival of T cells, unlike its apparent role in macrophages [54]. T cells do not depend on HIF-1α for survival to the same degree as macrophages since activated T cells produce ATP by both glycolysis and oxidative phosphorylation [55]. Rather, HIF-1α in T cells appears to play an anti-inflammatory and tissue-protecting role by negatively regulating T cell functions [52,56,57]. Indeed, T cell-targeted disruption of HIF-1α leads to increased IFN-γ secretion and/or improved effector functions [58-61]. Although available data on gene expression profiles in Th1 and Th2 cells do not suggest differential expression of HIF-1α mRNA between these cell populations [62], as is often the case in miR-mediated gene expression regulation, miR-17-92 may still regulate HIF-1α protein expression at post-transcriptional levels. These data collectively suggest that miR-17-92 expression in activated T cells may promote the type-1 function of T cells at least partially through down-regulation of HIF-1α.

The human Jurkat T cell line with ecotopic expression of miR-17-92 cluster members demonstrate increased IL-2 production and improved viability following treatment with the AICD condition (Fig. 6). The Jurkat cell line was established from the peripheral blood of a T cell leukemia patient in the 1970s. This cell line is often used to recapitulate what would happen in humans T cells as the line retains many T cell properties, such as CD4, a T cell receptor, and ability to produce IL-2 [63]. For these reasons, we chose to use Jurkat cells in our experiments. We recognize, on the other hand, that this cell line has pitfalls since this is a tumor cell line with enhanced survival compared to normal T cells due to their intrinsic biology. Thus, continued work with human T cells is clearly warranted.

miRs in the miR-17-92 clusters are amplified in various tumor types including B cell lymphoma and lung cancer, and promote proliferation and confer anti-apoptotic function in tumors, thereby promoting tumor-progression and functioning as oncogenes [27-31]. However, miR-17-92 by itself may not be responsible for oncogenesis as transgenic mice with miR-17-92 overexpressed in lymphocytes develop lymphoproliferative disorder and autoimmunity but not cancer [24]. miR-17-92 may cooperate with other oncogenes to promote the oncogenic process. Transgenic mice overexpressing both miR-17-92 and c-Myc in lymphocytes develop early onset lymphomagenesis disorders [27]. On the other hand, knockout studies of the miR-17-92 cluster in mice have demonstrated the importance of this cluster in mammalian biology. While knockout of the miR-17-92 cluster results in immediate post-natal death of all progeny, knockout of either or both the miR-106a or miR-106b clusters are viable without an apparent phenotype [64]. However knock out of the miR-17-92 cluster together with miR-106a or 106b cluster results in embryonic lethality [25].

During lymphocyte development, miR-17-92 miRs are highly expressed in progenitor cells, with the expression level decreasing 2- to 3-fold following maturation [24]. In addition, we have evaluated relative expression of miR-17-92 in a variety of Th cells as well as naïve CD4^+ ^cells. Naïve CD4^+ ^cells express miR-17-92 at the highest level among the cell populations examined. Albeit lower than that in naïve CD4^+ ^cells, Th1 cells express miR-17-92 at higher levels than T neutral (anti-CD3, feeder cells and IL-2) and Th17 cells, and Th2 cells consistently exhibit the lowest levels of miR-17-92 among the populations tested (data not shown). More studies are warranted on the specific role of miR-17-92 during differentiation.

These studies reviewed above provide us with critical insights as to what has to be expected if we develop therapeutic strategies by modulating miR-17-92 expression. One major barrier for successful T cell-based cancer immunotherapy is the low persistence of tumor antigen (TA)-specific T cells in tumor-bearing hosts [65,66]. It seems promising to generate genetically modified TA-specific T cells *ex vivo *that are resistant to tumor-mediated immune suppression and mediate robust and long-lived anti-tumor responses. miR-17-92 cluster has the potential to confer resistance to tumor-derived immunosuppressive factors and to improve type-1 reactivity. Further characterization of the role of miR-17-92 cluster in tumor antigen (TA)-specific CTLs is clearly warranted and may provide us with ability to develop novel immunotherapy strategies with genetically engineered T cells. Additionally, identification of diminished miR-17-92 expression in the peripheral blood may emerge as an important biomarker in patients with malignancy.

## Abbreviations

miR: microRNA; VLA-4: very late antigen-4; AICD: apoptosis induced cell death; CNS: central nervous system; GBM: glioblastoma multiforme; ICAM-1: intracellular cell adhesion molecule-1; STAT6: signal transducers and activators of transcription-6; PMA: phorbol myristate acetate; SPC: splenocyte.

## Competing interests

The authors declare that they have no competing interests.

## Authors' contributions

GK participated in the conception of the study, experimental design, performed *in vivo *and *in vitro *assays, and was one of the two primary writers of the paper. KS was involved in the conception of the study, further designing of the experiments, and took a primary role in culturing the differentiated T cells. AH performed studies using Jurkat cells. MF participated in experimental design, troubleshooting, editing the manuscript and statistical analysis. RU assisted in microRNA array and expression studies and analysis. HM helped with the ELISA and technical editing of the manuscript. TAR, JM and MTL participated in the design of experiments and interpretation of data. EW and FMM performed the miR microarray and assisted with analysis. HO conceived the study, mentored primary authors, was one of the two primary writers of the paper, and heavily participated in experimental design and data analysis. All authors read and approved the final manuscript.
